# University life and pandemic influenza: Attitudes and intended behaviour of staff and students towards pandemic (H1N1) 2009

**DOI:** 10.1186/1471-2458-10-130

**Published:** 2010-03-14

**Authors:** Debbie Van, Mary-Louise McLaws, Jacinta Crimmins, C Raina MacIntyre, Holly Seale

**Affiliations:** 1 Faculty of Medicine, The University of New South Wales, Australia; 2 School of Public Health and Community Medicine, Faculty of Medicine, The University of New South Wales, Australia; 3 University Health Service, The University of New South Wales, Australia; 4 National Centre for Immunisation Research and Surveillance of Vaccine Preventable Diseases (NCIRS), The Children's Hospital at Westmead and Discipline of Paediatrics and Child Health, University of Sydney, New South Wales, Australia

## Abstract

**Background:**

In a pandemic young adults are more likely to be infected, increasing the potential for Universities to be explosive disease outbreak centres. Outbreak management is essential to reduce the impact in both the institution and the surrounding community. Through the use of an online survey, we aimed to measure the perceptions and responses of staff and students towards pandemic (H1N1) 2009 at a major university in Sydney, Australia.

**Methods:**

The survey was available online from 29 June to 30 September 2009. The sample included academic staff, general staff and students of the University.

**Results:**

A total of 2882 surveys were completed. Nearly all respondents (99.6%, 2870/2882) were aware of the Australian pandemic situation and 64.2% (1851/2882) reported either "no anxiety" or "disinterest." Asian-born respondents were significantly (p < 0.001) more likely to believe that the pandemic was serious compared to respondents from other regions. 75.9% (2188/2882) of respondents had not made any lifestyle changes as a result of the pandemic. Most respondents had not adopted any specific behaviour change, and only 20.8% (600/2882) had adopted the simplest health behaviour, i.e. hand hygiene. Adoption of a specific behaviour change was linked to anxiety and Asian origin. Students were more likely to attend the university if unwell compared with staff members. Positive responses from students strongly indicate the potential for expanding online teaching and learning resources for continuing education in disaster settings. Willingness to receive the pandemic vaccine was associated with seasonal influenza vaccination uptake over the previous 3 years.

**Conclusions:**

Responses to a pandemic are subject to change in its pre-, early and mid-outbreak stages. Lessons for these institutions in preparation for a second wave and future disease outbreaks include the need to promote positive public health behaviours amongst young people and students.

## Background

In April 2009, severe cases of pneumonia preceded by influenza-like illness were noted to occur in Mexico and then North America. A novel influenza A (H1N1) virus was identified as the cause and it rapidly evolved into a pandemic. Cases of the strain were first identified in Australia in early May and soon appeared across the country [1]. As of February 12 2010, there have been 37,000 confirmed cases and 191 deaths in Australia. While this pandemic has been moderate or milder than previous pandemics such as the Spanish flu of 1918-1919, similarities can be drawn between the two in regards to the median age of cases. In Australia, the median age of confirmed cases is 21 years [2]

Universities therefore have the potential to become explosive, centrifugal outbreak centres due to their large young adult population, high levels of close social contact and permeable boundaries. During a pandemic or disease outbreak, the proportion affected may exceed the seasonal norm of one-third of the student population [3]. As sites of transmission, they may have a negative impact on the larger communities in which they are embedded. Additionally, student behaviour is often divergent from non-student adult populations [4]. Hence, understanding of and outbreak management in such institutions are essential to minimise the impact of pandemic influenza in both the institution and its surrounds.

University settings are unique given the permeability of their boundaries and the groups, and the activities within the institution that affect social contact between its members. Both of which have the potential to affect behaviours and perceptions. This survey was conducted to examine the understanding of and attitudes towards pandemic (H1N1) 2009 amongst students and staff at the University of New South Wales (UNSW), Sydney, Australia and their behavioural intentions during this pandemic.

## Methods

### Context

The CDC recommends that institutions of higher education balance the goals to minimize morbidity and mortality from pandemic influenza with the goal of minimising educational and social disruption [5]. Between April 30^th ^and September 30^th ^2009, ten broadcast emails were sent out by the Director of the University Health Service to staff and students. Contained in the emails was information on the: (1) H1N1 situation; (2) modes of spread and common symptoms; (3) recommended health advice consistent with both the WHO and national recommendations; and (4) contact information for the relevant health departments [6]. Posters developed by the Commonwealth Department of Health and Ageing and UNSW were placed in high traffic areas and focused on: (1) encouraging faculty, staff and students to stay at home if symptomatic (i.e. with a fever, cough, and runny nose) and to protect each other; (2) cough/sneeze etiquette (i.e. "cover your mouth and nose when you cough and sneeze" and "dispose of used tissues in the bin) and (3) hand hygiene (i.e. "Wash your hands properly and regularly").

### Survey

Data was collected from the 29 June - 30 September, coinciding with the peak of the pandemic in Australia. An anonymous online survey was designed to assess the knowledge, attitudes and perceptions of pandemic (H1N1) 2009, which was referred to by its vernacular alternative, "swine flu". The survey was piloted on June 11th with three students and three staff, representative of the members of the study population, and modified accordingly. The final version assessed: (1) demographic characteristics; (2) awareness, perceived personal risk and anxiety; (3) recent influenza-related behaviours changes; (4) intended behaviour in the event of various scenarios at UNSW and (5) compliance with different community interventions. In regards to the behaviour changes, we included four changes (cancelling social plans, avoiding busy public places, cancelling/postponing travel plans and not using public transport) that were related to avoidance behaviour and not recommended by the government and five changes (buying hygiene products, receiving the seasonal influenza vaccine, using online resources for teaching and learning and stockpiling necessities) that were related to recommended behaviours. The recommended behaviours questions were adapted with permission from a study undertaken by Rubin et al [7].

### Participants

The sample comprised of both academic and non-academic staff (i.e. administration, IT and other support staff) and students at UNSW in Sydney, Australia. Participants accessed the final questionnaire via a link on an online newsletter available to all UNSW staff and students, and via an online information gateway. Emails were also sent to the heads of each Faculty, informing them of the survey. Consent was implied upon completion and submission of the questionnaire. Submitted surveys were collated in a directory and de-identified prior to analysis. Participants were offered the chance to win a $500 cash prize upon completing the survey. Ethics approval was granted by the University of New South Wales Human Research Ethics Committee.

### Analysis

The quantitative data on the completed survey was collected in Microsoft Excel. OpenEpi (version 2.3) was used to calculate [8], proportions, 95% confidence intervals and χ^2 ^tests for significance. Alpha was set at the 5% level. We used logistic regression to compute odds ratios to evaluate the association of demographic variables and attitudes and beliefs.

## Results

### Participants

A total of 2882 UNSW staff and students aged ≥18 years completed the online survey between the 29 June and 30 September 2009. The overall response rate was 5.7% (2882/50847) and most respondents were young (18-34 years, 73.9%, 2129/2882) and born in Australia (51.8%, 1492/2882) (Table 1). Academic and general staff members were both overrepresented in our sample (12.5%, 360/2882, X^2 ^= 311.3, p < 0.001; 22.4%, 646/2882, X^2 ^= 1313, p < 0.001 respectively) compared to the actual proportions employed at UNSW (4.9%, 2497/50847 and 5.5%, 2779/50847 respectively). Students were underrepresented (65.1%, 1876/2882, X^2 ^= 1279, p < 0.001) compared to the proportion of internal students at UNSW (88.2%, 44833/50847).

**Table 1:** Demographic characteristics of the participants

Characteristic	Academic Staff	General Staff	Students	Total
	n = 360 (%)	n = 646 (%)	n = 1876 (%)	n = 2882 (%)
**Sex**				
Male	193 (53.6)	199 (30.8)	914 (48.7)	1306 (45.3)
Female	162 (45.0)	429 (66.4)	930 (49.6)	1521 (52.8)
Not specified	5 (1.4)	18 (2.8)	32 (1.7)	55 (1.9)
**Age group (years)**				
18-24	29 (8.1)	65 (10.1)	1393 (74.3)	1487 (51.6)
25-34	88 (24.4)	191 (29.6)	363 (19.3)	642 (22.3)
35-44	93 (25.8)	151 (23.4)	65 (3.5)	309 (10.7)
45-54	80 (22.2)	150 (23.2)	38 (2.0)	268 (9.3)
55-64	57 (15.8)	84 (13.0)	11 (0.6)	152 (5.3)
≥ 65	13 (3.6)	2 (0.3)	1 (0.1)	16 (0.6)
Not specified	0 (0.0)	3 (0.5)	5 (0.3)	8 (0.3)
**Home/living arrangements**				
Live alone	41 (11.4)	97 (15.0)	116 (6.2)	254 (8.8)
Live with parents	25 (6.9)	71 (11.0)	846 (45.1)	942 (32.7)
Live with partner/spouse	126 (35.0)	192 (29.7)	197 (10.5)	515 (17.9)
Live in shared accommodation	27 (7.5)	76 (11.8)	516 (27.5)	619 (21.5)
Other	141 (39.2)	210 (32.5)	101 (5.4)	452 (15.7)
Not specified	0	0	100 (5.3)	100(3.4)
**Country of birth**				
Australia	178 (49.4)	386 (59.8)	928 (49.5)	1492 (51.8)
Asia	42 (11.7)	82 (12.7)	628 (33.5)	752 (26.1)
Europe	75 (20.8)	90 (13.9)	149 (7.9)	314 (10.9)
Other	65 (18.1)	88 (13.6)	171 (9.1)	324 (11.2)
**Time spent in Australia**				
≤ 2 years	30 (8.3)	22 (3.4)	311 (16.6)	363 (12.6)
3-5 years	24 (6.7)	26 (4.0)	206 (11.0)	256 (8.9)
6-10 years	32 (8.9)	38 (5.9)	117 (6.2)	187 (6.5)
>10 years	265 (73.6)	548 (84.8)	1214 (64.7)	2027 (70.3)
Not specified	9 (2.5)	12 (1.9)	28 (1.5)	49 (1.7)
**Employment**				
Unemployed	0	0	820 (43.7)	820 (28.5)
Full time	274 (76.1)	493 (76.3)	200 (10.7)	967 (33.6)
Casual	26 (7.2)	52 (8.0)	567 (30.2)	645 (22.3)
Part-time	50 (13.9)	94 (14.6)	277 (14.8)	421 (14.6)
Not specified	10 (2.8)	7 (1.1)	12 (0.6)	29 (1)

### Knowledge, attitude and perceptions

**Figure 1:**
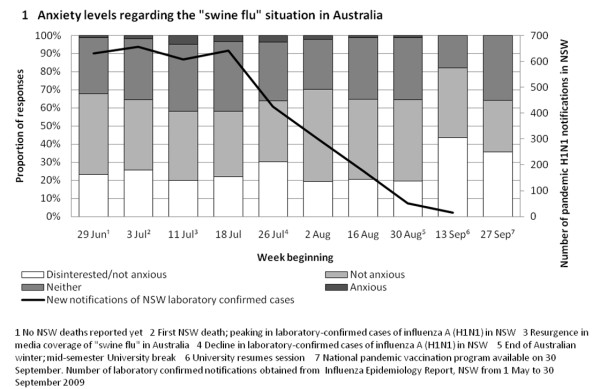
**Attitudes towards pandemic (H1N1) 2009 amongst University staff and students**.

**Figure 2:**
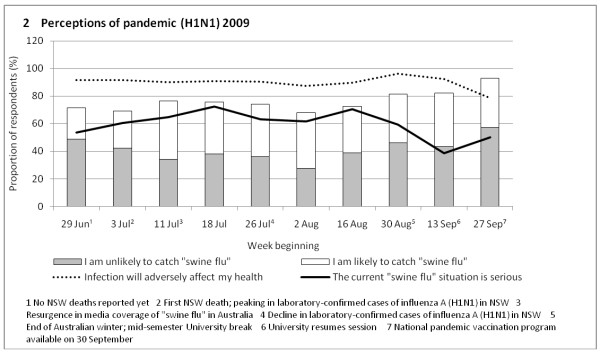
**Perceptions of pandemic (H1N1) 2009 amongst University staff and students**.

Most respondents (99.6%, 2870/2882) reported that they had heard about the Australian pandemic situation. Whilst 60.4% (1742/2882) believed that it was serious, 40.4% (1165/2882) said that they were "not anxious" (Figure 1) and a further 23.8% (686/2882) reported "disinterest". Of the respondents who felt they were likely to contract pandemic influenza (42.2%, 1217/2882), 90.7% (1104/1217) believed the infection would adversely affect their health. Towards the end of the survey period and the end of winter, the percentage reporting "no anxiety" increased and the proportion of respondents who believed that the pandemic was "serious" significantly decreased (OR, 0.25 [95% CI, 0.17-6.38); p < 0.001) (Figure 2). Perceptions of susceptibility significantly decreased with the decline of laboratory-confirmed cases in Australia (OR, 0.72 [95% CI, 0.57-0.92]; p = 0.003). Asian-born respondents were significantly more likely to believe that the pandemic was serious (OR, 3.79 [95% CI, 3.16-4.55]; p < 0.001) and feel anxious (OR, 4.85 [95% CI, 2.94-8.13]; p < 0.001) compared with respondents from all other regions. Females were significantly more likely than males to think their health would be adversely affected (OR, 1.78 [95% CI, 1.37-2.33]; p < 0.001), and that the current situation is serious (OR, 1.21 [95% CI, 1.04-1.40]; p = 0.01). Respondents aged over 55 were most likely to be anxious (OR, 2.28 [95% CI, 1.00-4.68]; p = 0.02) and younger respondents aged 20-34 were the least likely group to believe that they were susceptible to infection (43.7%, 930/2129, p = 0.04).

### Lifestyle and specific behavioural changes

Overall, most respondents (75.9%, 2188/2882) had not made any lifestyle changes, and 61.8% (1781/2882) had not undertaken any specific health behaviours due to the pandemic (Table 2). The most commonly reported change was the purchase of hygiene products such as face masks or hand hygiene products (20.8%, 600/2882). Asian-born respondents were significantly more likely than Australian-born respondents to indicate that they had made a lifestyle change (OR, 2.78 [CI, 2.26-3.39]; p < 0.01), although this likelihood decreased with time spent in Australia. Uptake of avoidant behaviour was significantly related to being a student (32.1%, 603/1876, X^2 ^= 44.14, p < 0.001) and belief that infection would have adverse health effects (OR, 2.22 [CI, 1.07-5.24]; p = 0.02). Those who had made at least one recommended behaviour change were significantly more likely to be anxious about the pandemic (OR, 4.27 [CI, 1.61-11.10]; p = 0.002), and were female (OR, 1.46 [CI, 1.15-1.84]; p = 0.001).

**Table 2:** Behavioural responses to pandemic (H1N1) 2009

Indicator	Academic Staff	General Staff	Students	Total
	n = 360 (%)	n = 646 (%)	n = 1876 (%)	n = 2882 (%)
**Have you made changes to the way you live your life because of the current "swine flu situation"?**				
Yes	102 (28.3)	156 (24.1)	436 (23.2)	694 (24.1)
No	258 (71.7)	490 (75.9)	1440 (76.8)	2188 (75.9)
				
**Have you undertaken any of the following because of "swine flu"?**				
*Avoidant*				
I have cancelled plans to meet friends, other students, family members	12 (3.3)	20 (3.1)	100 (5.3)	132 (4.6)
I have avoided busy public places e.g. shopping areas, cinemas, restaurants	31 (8.6)	67 (10.4)	261 (13.9)	359 (12.5)
I have cancelled/postponed travel plans-interstate or international	22 (6.1)	26 (4.0)	170 (9.1)	218 (7.6)
I have stopped using public transport	13 (3.6)	25 (3.9)	72 (3.8)	110 (3.8)
				
*Non-disruptive*				
I have bought hygiene products-face masks, hand wash to protect me from swine flu	76 (21.1)	132 (20.4)	392 (20.9)	600 (20.8)
I have gotten a flu shot to help protect me swine flu from	59 (16.4)	102 (15.8)	134 (7.1)	295 (10.2)
I have switched to listening to lecturers/participating in tutorials online	1 (0.3)	3 (0.5)	26 (1.4)	30 (1.0)
I have organised to record lectures/tutorials	2 (0.6)	1 (0.2)	18 (1.0)	21 (0.7)
I have stocked up on food and/or other necessities	20 (5.6)	23 (3.6)	74 (3.9)	117 (4.1)
				
*No behaviour change*				
I haven't changed any of my behaviour/plans	207 (57.5)	414 (64.1)	1160 (61.8)	1781 (61.8)

### Compliance with specific measures

60.2% (1736/2882, p < 0.001) of respondents were willing to comply with all five public health measures if requested by authorities (Table 3). Asian-born respondents again had the highest rates (70.6%, 531/752, X^2 ^= 45.73, p < 0.001). Willingness to receive the pandemic vaccine (despite it being unavailable at the time of survey) was associated with seasonal influenza vaccination uptake over the previous 3 years, compared with those who had no vaccination history (99.1% v 85.7%, X^2 ^= 32.11, p < 0.001). The use of face masks in public was met with most resistance (16.8%, 484/2882), especially amongst respondents aged 18-24 years (19.5%, 290/1487, p < 0.001).

**Table 3:** Intended compliance with specific public health measures

Specific public health measure	Academic Staff	General Staff	Student	Total
	n = 360 (%)	n = 646 (%)	n = 1876(%)	n = 2882 (%)
**Receive a "swine flu" vaccine**				
Reluctant	18 (5.0)	49 (7.6)	100 (5.3)	167 (5.9)
Willing	102 (28.3)	242 (37.4)	610 (32.5)	954 (33.1)
Very willing	230 (63.9)	332 (51.4)	1107 (59.0)	1669 (57.9)
NA	3 (0.8)	2 (0.3)	5 (0.3)	10 (0.3)
Not specified	7 (1.9)	21 (3.3)	54 (2.9)	82 (2.8)
**Isolation from others if infected with "swine flu"**				
Reluctant	3 (0.8)	6 (0.9)	83 (4.4)	92 (3.2)
Willing	74 (20.6)	104 (16.1)	605 (32.2)	783 (27.2)
Very willing	280 (77.8)	533 (82.5)	1149 (61.2)	1962 (68.1)
NA	1 (0.3)	1 (0.2)	6 (0.3)	7 (0.2)
Not specified	2 (0.6)	2 (0.3)	33 (1.8)	38 (1.3)
**Wear a mask in public if experiencing flu-like symptoms**				
Reluctant	41 (11.4)	95 (14.7)	348 (18.6)	484 (16.8)
Willing	163 (45.3)	301 (46.6)	899 (47.9)	1363 (47.3)
Very willing	149 (41.4)	225 (34.8)	581 (31.0)	955 (33.1)
NA	0	8 (1.2)	8 (0.4)	16 (0.6)
Not specified	7 (1.9)	17 (2.6)	40 (2.1)	64 (2.2)
**Assist quarantined neighbours/friends with food supplies etc**				
Reluctant	5 (1.4)	8 (1.2)	86 (4.6)	99 (3.5)
Willing	76 (21.1)	174 (26.9)	730 (38.9)	980 (34.0)
Very willing	276 (76.7)	454 (70.3)	989 (52.7)	1719 (59.6)
NA	0	3 (0.5)	7 (0.4)	10 (0.3)
Not specified	3 (0.8)	7 (1.1)	64 (3.4)	74 (2.6)
**Participated by staying away and taking lectures/tutorials via**				
**the internet**				
Reluctant	13 (3.6)	9 (1.4)	119 (6.3)	141 (4.9)
Willing	94 (26.1)	88 (13.6)	715 (38.1)	897 (31.1)
Very willing	181 (50.3)	317 (49.1)	974 (51.9)	1472 (51.1)
NA	62 (17.2)	229 (35.4)	32 (1.7)	323 (11.2)
Not specified	10 (2.8)	3 (0.5)	36 (1.9)	49 (1.7)

### Intended behaviour at UNSW

Just over half (51.1%, 1472/2882), were very willing to continue University schooling online however, academic staff were significantly more reluctant than students to deliver teaching by this method (OR, 0.56 [CI, 0.30-0.97]; p = 0.02). 78.5% (2282/2882) of respondents would not come to university if they were experiencing influenza-like illness (ILI) (Table 4). Students were the most likely to attend University if unwell (75%, 1407/1876, p < 0.001) and were the only group where over half would present if they had an assessment or deadline due and were unwell (66.5%, 1248/1876). However respondents who had made a lifestyle change as a result of pandemic (H1N1) were significantly more likely to absent themselves even if an exam or deadline was due (X^2 ^= 50.45, p < 0.001).

**Table 4:** Intended behaviour at the University

"I would come into university if..."	Academic Staff	General Staff	Students	Total
	n = 360 (%)	n = 646 (%)	n = 1876 (%)	n = 2882 (%)
**I had symptoms consistent with flu, e.g. fever and a cough/runny nose**	57 (15.8)	86 (13.3)	407 (21.7)	550 (19.1)
**I had symptoms consistent with flu and I had an exam/assessment due or work deadline**	155 (43.1)	256 (39.6)	1248 (66.5)	1659 (57.6)
**I was well but knew that a fellow student or staff member had contracted the flu**	295 (81.9)	549 (85.0)	1428 (76.1)	2272 (78.8)
**A family member or close contact had flu-like symptoms e.g. fever and a cough/runny nose**	269 (74.7)	524 (81.1)	1451 (77.7)	2244 (77.9)
**I was well but I knew that the number of cases of swine flu had increased in Australia**	341 (94.7)	612 (94.7)	1719 (91.6)	2672 (92.7)

**"If you attend a lecture/meeting and are sitting near someone who is coughing/sneezing, would you."**				

**Not be worried**	67 (18.6)	108 (16.7)	366 (19.5)	541 (18.8)
**Approach the individual**	5 (1.4)	4 (0.6)	3 (0.2)	12 (0.4)
**Approach the individual and ask him/her to comply with specific health measures**, e.g. leaving the area, observing respiratory etiquette	23 (6.4)	61 (9.4)	60 (3.2)	144 (5.0)
**Not approach the individual**	118 (32.8)	225 (34.8)	776 (41.4)	1119 (38.8)
**Not approach the individual and increase personal hygiene**, e.g. moving away, leaving the area or hand-washing afterwards	144 (40.0)	244 (37.8)	665 (35.4)	1053 (36.5)

## Discussion

Universities are not immune to natural or manmade disasters, and past experience with these have illustrated the importance of continuity during and after these events [5,9]. In an influenza pandemic, such institutions must maintain a balance between academic continuity, with infection control and minimising morbidity [5].

In contrast to pre-pandemic and early pandemic findings in Australian communities [10,11], most of the University population surveyed were not anxious about the Australian pandemic situation nor did they think it was serious. Younger respondents (aged 20-34) were most likely to believe they were not susceptible to pandemic H1N1 2009, despite being the most affected group in previous influenza pandemics. Following the resurgence of media coverage of "swine flu" in Australia, we did measure a significant rise in anxiety, perceived susceptibility and seriousness. This however declined with the approach of spring and the decline of laboratory confirmed cases of influenza A (H1N1) in NSW [12]. This illustrates that public perception of a pandemic is unstable, especially when the severity and natural progression cannot be accurately predicted.

If requested by authorities, most respondents in our cohort were willing to undergo isolation if suffering from influenza-like-illness (ILI). Of concern was the high proportion of students who indicated that they would still attend University with symptoms. In the event of an exam or assessment deadline, their proportion tripled. Such behaviour is detrimental for both students and the community, for in addition to spreading the pandemic virus, students with ILI are also likely have reduced academic performance by up 30-60% [3]. Absenteeism from University was higher in respondents who had indicated making a lifestyle change, implying the practicality of encouraging general positive health behaviour in this population. Along with encouraging students to self-isolate in the case of illness, there must be ongoing education about the importance of infection control, especially when anxiety rates and risk perceptions are low. Health messages need to educate students about the impact of the illness on their studies, and Universities should emphasise their illness/misadventure assessment policies during disease outbreaks.

Online resources such as lecture recordings and forum tutorials allow for off-campus education, and can provide continuity of learning for students undergoing isolation. However in our study, few respondents had adopted the use of online teaching or learning resources as a result of pandemic influenza (H1N1). This may be due to a number of factors including: (1) the apparent mildness of the pandemic; and/or (2) the lack of promotion by the University to use these resources. It was encouraging to see that students were very willing to continue University schooling via online resources, indicating the potential for expanding the existing UNSW online teaching resources. While it was encouraging that students would undertake online courses, we found very little support for an online teaching method among the academic staff members. Reluctance to use online resources was associated with increased age, and may be due to unfamiliarity with or resistance to technology. In preparation for an outbreak, Universities should focus on creating additional support for technologies that allow faculty and students to continue their teaching and learning activities which minimise disruption. Online recordings, virtual learning environment, blogs, web conferencing and discussion forums should all be utilised to assist in the delivery of lessons. Having a contingency and communication plan for teaching key sections may provide the needed continuity for students and faculty. Training must be provided in the pre-pandemic periods to minimise disruption.

We found that most respondents had not made any lifestyle changes or undertaken any specific behaviour change despite receiving information from the University. This may be attributed to the mildness of pandemic (H1N1) 2009. This finding supports both the pre-pandemic and post-SARS findings on the dose-response relationship between outbreak severity and the responses to it [13,14]. Of the respondents who did indicate behaviour change, increased hand hygiene was the most common. It would therefore be beneficial and at minimal cost for institutions such as Universities to provide extra hand-washing facilities and posters encouraging compliance in communal areas and computer labs. Universities could also boost hygienic practices by openly distributing small bottles of hand gels or tissue packets to staff and students on campus.

Close to 60% of our respondents stated that they were 'very willing' to receive a hypothetical pandemic vaccine. As the survey period ended before the vaccine became available, we were unable to follow up participants to ascertain if they did receive the vaccine. In Australia, the H1N1 vaccine was not released until September 2009, by which time, virus activity was very low. A recent national survey [15], found that although 96% of the Australian cohort was aware of an available pandemic vaccine, less than 20% had received the vaccine. We can therefore expect similarly low vaccination rates in our cohort. The survey also identified that uptake of the H1N1 vaccine was three times as high in those aged 65 years and over (42%) than in those aged 18-64 years (14%), with no statistically significant difference between males and females [15]. We found that respondents who had received seasonal influenza vaccinations in the past were significantly more likely to accept the pandemic vaccine then their non-vaccinated counterparts. These findings are consistent with several recent studies on pandemic vaccine uptake [16,17]. Providing the vaccine through clinics or university health facilities should help bolster vaccine uptake, especially for international students, who may not have access to free healthcare.

Of the participants surveyed, Asian-born respondents were the most likely to be anxious about the Australian pandemic situation, rate the situation as serious, undertake specific behavioural changes and comply with public health measures. It could be hypothesised that these respondents, their families, friends or members of their communities may have been exposed to previous infectious disease situations such as SARS and avian influenza [18,19]. If not exposed, at the least these respondents have lived in countries where their governments have had to deal with these situations, leading to stricter infection control standards and higher levels of media exposure. Interestingly, Asian born respondents who have been settled in Australia for longer periods were less likely to have made any lifestyle changes compared to their counterparts who have been in the country for only short amount of time. It would appear that living in Australia dilutes the tendency to adopt behavioural changes, and it would be beneficial for future studies to identify aspects of Australian culture which influence health behaviours.

We acknowledge that this study has several limitations. These include: (1) the survey was restricted to the UNSW student, general and faculty staff, mostly highly educated Sydney residents; (2) the electronic format of the survey may have excluded persons without internet access; (3) we did not defin what "requested by the authorities meant" so it was open to the respondents interpretation and (4) the survey was not translated into other languages. However, English is the dominant language used in both teaching and communication and UNSW relies heavily on electronic communication with its campus population to disseminate other unrelated information in English. There was no established measure of influenza protective behaviour, as most of the survey items were developed prior to the publication of the CDC Guidance for Responses to Influenza for Institutions of Higher Education [5]. The declining number of participants who accessed the online survey towards the end of the survey period likely restricted analysis of how responses to the pandemic change over time.

## Conclusions

From the study results, several key messages should be drawn. Firstly, risk perceptions and anxiety are low and will remain so unless there is a major shift in the virus. This will continue to impact on compliance or uptake of mitigation strategies. Secondly, more effective health communication and management is needed to promote self-isolation and infection control in the event of illness especially amongst students. These students are unlikely to adopt behaviours that are unknown to them. Therefore the focus should be on handwashing and cough etiquette. Lastly, universities must invest in online teaching resources and training during inter-pandemic periods. There also needs to be greater recognition for the need for online assignment submission and examinations to ensure minimal disruption to the students.

## Competing interests

Raina MacIntyre receives funding from influenza vaccine manufacturers GSK and CSL Biotherapies for investigator-driven research. These payments were not associated with this study. The remaining authors have no competing interests.

## Authors' contributions

DV/HS participated in the design of the study and survey, undertook the distribution and collection, performed the analysis and drafted the manuscript. MLM participated in the design of the study and survey, assisted with the analysis and reviewed the manuscript. JC/CRM participated in its design and coordination and helped to draft the manuscript. All authors read and approved the final manuscript.

## Pre-publication history

The pre-publication history for this paper can be accessed here:

http://www.biomedcentral.com/1471-2458/10/130/prepub
